# Correction: Combining genetic and demographic monitoring better informs conservation of an endangered urban snake

**DOI:** 10.1371/journal.pone.0257374

**Published:** 2021-09-09

**Authors:** Dustin A. Wood, Jonathan P. Rose, Brian J. Halstead, Ricka E. Stoelting, Karen E. Swaim, Amy G. Vandergast

In the Author Contributions, Ricka Stoelting (RS) should be listed as one of the persons who contributed to methodology and performed formal analysis.

The range of dates provided in the Competing Interests statement is incorrect. The correct Competing Interests statement is: Swaim Biological, Inc collected *Thamnophis sirtalis tetrataenia* tissues between the years 2005–2018 that were provided as an in-kind service and these tissues were utilized in this study. Site data from a previously published study (Reeder et al., 2015), which was partially funded by Swaim Biological, Inc, were also used in the present study. This does not alter our adherence to PLOS ONE policies on sharing data and materials. There are no patents, products in development or marketed products associated with this research to declare.
