# Supplementary material for: Combining genetic and demographic monitoring better informs conservation of an endangered urban snake
Source: PLoS One. 2020 May 5;15(5):e0231744. doi: 10.1371/journal.pone.0231744 (PMC7200000; doi:10.1371/journal.pone.0231744)
Supplement: S1 Fig — (PDF) [file pone.0231744.s001.pdf]

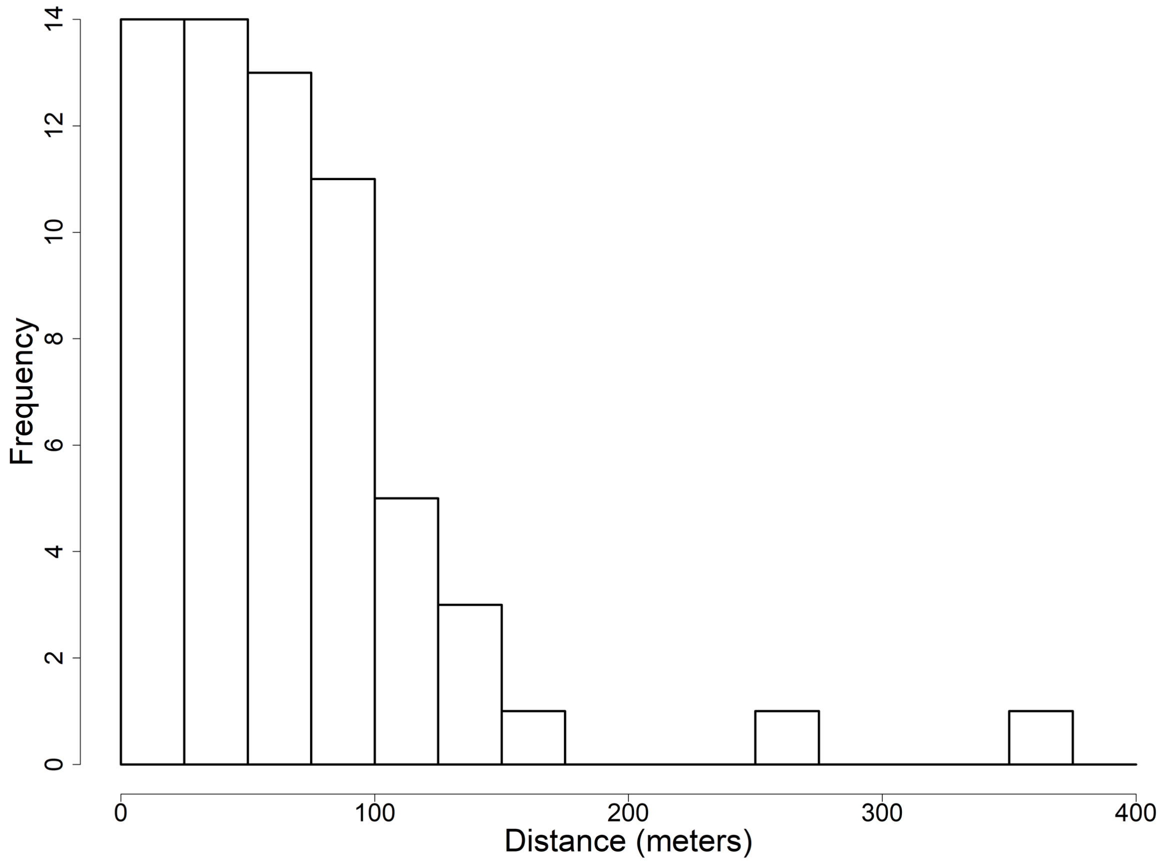

S1 Figure. Frequency histogram of distances moved by San Francisco gartersnakes (*Thamnophis sirtalis tetrataenia*) between captures at five sites sampled in 2018.
