# Supplementary material for: Combining genetic and demographic monitoring better informs conservation of an endangered urban snake
Source: PLoS One. 2020 May 5;15(5):e0231744. doi: 10.1371/journal.pone.0231744 (PMC7200000; doi:10.1371/journal.pone.0231744)
Supplement: S2 Fig — (PDF) [file pone.0231744.s002.pdf]

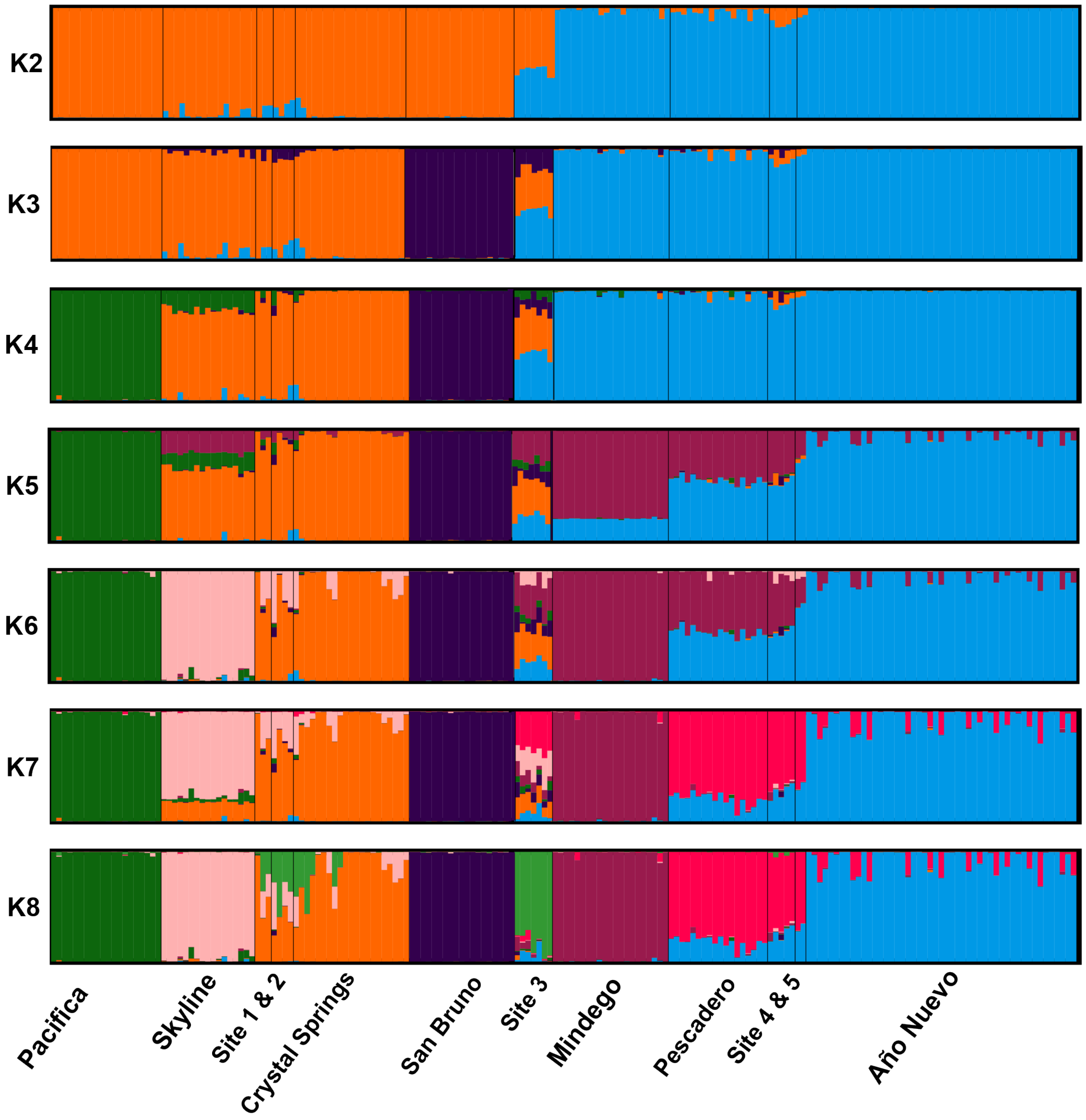

S2 Figure. STRUCTURE **assignments of individuals for K = 2-8 across all sites using the *T. s. tetrataenia* rangewide dataset.**
