# Supplementary material for: Combining genetic and demographic monitoring better informs conservation of an endangered urban snake
Source: PLoS One. 2020 May 5;15(5):e0231744. doi: 10.1371/journal.pone.0231744 (PMC7200000; doi:10.1371/journal.pone.0231744)
Supplement: S3 Fig — However, no meaningful patterns resulted from this analysis. (PDF) [file pone.0231744.s003.pdf]

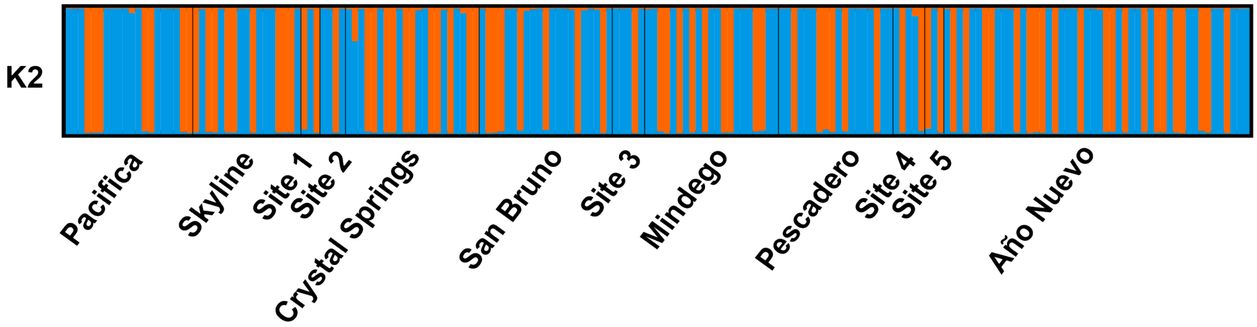

S3 Figure. **STRUCTURE assignment of individuals for K = 2 across all sites using the 12 putative outlier loci identified with BayeScan.** However, no meaningful patterns resulted from this analysis.
