# Supplementary material for: Combining genetic and demographic monitoring better informs conservation of an endangered urban snake
Source: PLoS One. 2020 May 5;15(5):e0231744. doi: 10.1371/journal.pone.0231744 (PMC7200000; doi:10.1371/journal.pone.0231744)
Supplement: S1 Table — For all sites we used a model that does not include individual heterogeneity in capture probability (p). Percentiles are lower and upper 95% Highest Posterior Density Interval limits. (PDF) [file pone.0231744.s004.pdf]

S1 Table: Priors for covariate effects on capture probability for sites sampled, listed by years sampled. For all sites we used a model that does not include individual heterogeneity in capture probability (p). Percentiles are lower and upper 95% Highest Posterior Density Interval limits.

**Year 2018**	**Sites: Año Nuevo, Cloverdale, Pacifica, Skyline, Crystal Springs**				**Percentile**	
**Parameter**	**Description**	**Prior**	**Median**	**SD**	**2.50%**	**97.50%**
p	Mean daily capture probability	Uniform(0,1)	0.016	0.004	0.008	0.025
βtemp	Effect of air temperature on p	Normal(0, 3.16)	0.02	0.078	-0.133	0.172
βSVL	Effect of snake SVL on p	Normal(0, 3.16)	0.228	0.126	-0.023	0.467
βsex	Effect of snake sex (male) on p	Normal(0, 3.16)	-0.185	0.273	-0.694	0.373
βtrap	Behavioral effect on p	Normal(0, 3.16)	-0.439	0.628	-1.775	0.66
σsite	SD of site random effect on p	half-Cauchy(1)	0.397	0.237	0.056	0.896
σt	SD of temporal random effect on p	half-Cauchy(1)	0.463	0.115	0.246	0.694
**Year 2017**	**Site: San Bruno**				**Percentile**	
**Parameter**	**Description**	**Prior**	**Median**		**2.50%**	**97.50%**
Logit(*p*)	Logit of mean daily capture probability	Normal(0, 31.6)	0.008		0.008	0.011
βsex	Effect of snake sex (female) on p	Normal(0, 31.6)T(-10,10)	-0.389		-0.693	-0.108
σt	SD of temporal random effect on p	Uniform(0,10)	0.582		0.326	0.874
**Year 2016**	**Site: Mindego**				**Percentile**	
**Parameter**	**Description**	**Prior**	**Median**	**SD**	**2.50%**	**97.50%**
p	Mean daily capture probability	Beta(1, 1)	0.005	0.002	0.002	0.01
βtemp	Effect of air temperature on p	Normal(0, 2)	0.378	0.2	0.000	0.787
βSVL	Effect of snake SVL on p	Normal(0, 2)	0.226	0.237	-0.243	0.684
βsex	Effect of snake sex (male) on p	Normal(0, 2)	0.369	0.393	-0.378	1.164
βtrap	Behavioral effect on p	Normal(0, 2)	2.121	0.384	1.362	2.871
σt	SD of temporal random effect on p	half-Cauchy(1)	0.878	0.181	0.562	1.259
