# Supplementary material for: Combining genetic and demographic monitoring better informs conservation of an endangered urban snake
Source: PLoS One. 2020 May 5;15(5):e0231744. doi: 10.1371/journal.pone.0231744 (PMC7200000; doi:10.1371/journal.pone.0231744)
Supplement: S2 Table — No values were significant using the α < 0.002 after Bonferroni correction. (PDF) [file pone.0231744.s005.pdf]

S2 Table. Pairwise genetic differentiation estimates using the 12 putative outlier loci identified with BayeScan for sampled sites with > 5 samples/site. No values were significant using the α < 0.002 after Bonferroni correction.

Sites	Pacifica	Skyline	Crystal Springs	San Bruno	Site 3	Mindego	Site 4	Pescadero
**Pacifica**	–							
**Skyline**	0.001	–						
**Crystal Springs**	0.002	0.000	–					
**San Bruno**	0.006	0.004	0.004	–				
**Site 3**	0.011	0.001	0.003	0.004	–			
**Mindego**	0.002	0.000	0.001	0.002	0.002	–		
**Site 4**	0.004	0.001	0.003	0.002	0.003	0.001	–	
**Pescadero**	0.004	0.004	0.006	0.005	0.008	0.000	0.006	–
**Año Nuevo**	0.003	0.000	0.000	0.002	0.001	0.000	0.002	0.003
