# Supplementary material for: Combining genetic and demographic monitoring better informs conservation of an endangered urban snake
Source: PLoS One. 2020 May 5;15(5):e0231744. doi: 10.1371/journal.pone.0231744 (PMC7200000; doi:10.1371/journal.pone.0231744)
Supplement: Attachment — Submitted filename: Response to Reviews_R1_R2.docx [file pone.0231744.s006.docx]

**Response to PlosOne Journal Reviews**

Reviewer #1: This paper represents a rigorous application of advanced genetic and demographic analyses in the context of a protected species and will contribute greatly to conservation of the focal subspecies. Data are presented in a clear and concise manner and all methods and results are explained fully. While not a consideration for publication in PLoS ONE, the topic and results of the study are both broadly and specifically interesting, presenting a valuable example of the use of the methods herein that can be applied elsewhere while also having practical implications for the management of a specific species.

I am recommending that the paper be accepted without revision. If revised, I provide the following recommendations and corrections.

Materials and Methods

Field methods and sample selection

Line 128 - describe tissue sampling. What samples were collected and how?

**Response to Reviewer 1: We added information regarding what type of tissue was taken and how this was conducted on page 6, lines 154-156.**

Line 128 - explain focal sites. How/why were focal sites chosen? How do focal sites differ from "satellite" sites? Is it just sample size?

**Response to Reviewer 1: We added text to help clarify the difference between focal & satellite sites with the section “Field methods and sample collection”. This can be found on page 6-7, lines 150-196. The focal sites were where we focused the demographic studies whereas satellite sites were not involved in demographic analyses and had much smaller sample sizes.**

Throughout - how do the sites compare to one another? In size? In habitat? In sampling coverage? It is mentioned in the discussion that some sites are much larger than the area that was sampled. Additional information to specify for which sites this was true and how it might affect the results or interpretation of the results would be helpful.

**Response to Reviewer 1:** **We have added estimates of the area effectively sampled by our traps for each site. Based on data we have on the distances moved by San Francisco gartersnakes between captures (97% of snake movements were < 200 m), we calculated the effective sample area for each site by buffering traps by 200 m and summing the area of habitat sampled by traps at each site. We have also added estimates of the total area of habitat available at each site (adding information in Table 1 and a supplemental figure, S1 Fig), so readers can compare how much of the habitat at each site was effectively sampled for San Francisco gartersnakes. The following text has been added to the methods (pages 8-9, lines 244–277):**

**“Sites differed in size of available habitat and in the area sampled by traps and cover objects. To define the total area of available habitat for each site, we created polygons in ArcGIS version 10.7.1 [33] that encompassed all suitable habitat, whether wetlands or non-forested uplands. The effective area sampled was then calculated by using a fixed buffer of 200 m around all trap and artificial cover object locations for each site. We chose a 200 m buffer based on the maximum distance moved between captures for greater than 95 percent of individual *T. s. tetrataenia* at our study sites (S1 Fig). The total site area and effective area sampled for each site are given in Table 1. At Pacifica and San Bruno, nearly all of the available habitat for *T. s. tetrataenia* was within the area effectively sampled by traps, and the nearest known population of *T. s. tetrataenia* was more than 2.5 km away. In contrast, although the area sampled at Skyline and Crystal Springs was comparable to Pacifica, only 60-70 percent of the area was sampled because suitable wetland habitat was present nearby and additional habitat (not included in our calculations) is present along most of the 7 km corridor separating these two sites. Año Nuevo, Mindego, and Pescadero were much larger sites and the area sampled was less than 60 percent of the available habitat.”**

A few grammatical errors:

Line 401 ..."filters we applied..." should be filters were applied

**Response to Reviewer 1: We fixed the grammatical error.**

Line 411 ..."where it was assigned as distinct genetic cluster..." should be assigned as a distinct genetic cluster

**Response to Reviewer 1: We fixed the grammatical error.**

Line 426 ..."each regional clusters that supported..." clusters should be cluster

**Response to Reviewer 1: We fixed the grammatical error.**

Reviewer #2: Wood et al. survey numerous sites for Thamnophis sirtalis tetrataenia and generate a RAD-seq data set for this taxon to better understand how best to implement future conservation efforts. I think that this is an interesting and important data set that has been thoroughly analyzed and has real world conservation applications. I have only a few comments listed below.

The sampling dates to look at temporal change in population genetic structure are 2004-2010 and 2015- 2018. How were these thresholds for change over time decided on? It seems arbitrary and not consistent across all sites.

**Response to Reviewer 2: This was an opportunistic use of previously collected, older samples that were available. We decided there were two breaks in time between concerted tissue sampling (1) an earlier temporal sample with a wider range of dates from 2004-2010 depending on the site and (2) a later temporal sample across a three year span 2015-2018.**

For the hierarchical structure analyses, was delta-K used in subsequent structure runs? If so, how was K=1 assessed (it is a known issue that with this particular method K=1 cannot be assessed)? Also I am assuming that the hierarchical structure analyses were run until there was no additional population structure? A bit more details would be good here.

**Response to Reviewer 2: Yes, we used delta-K in subsequent runs for structure analyses. As we mentioned in the methods, we evaluated the clustering results of our structure analyses using an independent analysis of population structure, discriminant analysis of principal components (DAPC). This analysis differs from structure analysis in that it does not require the assumptions of HWE. Given that we recovered the same clusters that were observed in the structure analysis, cluster results above K=1 are much more likely. In addition, the results from the mean log likelihood [lnP(D|K] score for each K value up to 7 or 8 had higher likelihood values than K at 1 (See Supporting information S2 Figure).**

L451 – Iʼm not entirely sold that this method is highlighting urban development as influencing population structure. Perhaps more details on how this is the case would clarify this. Otherwise, I think itʼs a bit overstated.

**Response to Reviewer 2: We removed the phrase “due to urban development” from the sentence. The sentence now reads as “In northern San Mateo County, cells separating San Bruno from other sites received “hotter’ values of genetic discontinuity, highlighting the isolation of individuals at this site.” See page 22, lined 639-641.**

Thatʼs super interesting that T. s. tetrataenia is paraphyletic given how morphologically different it is!

Whatʼs the longevity of these snakes – are the authors certain that in some of these small populations that the same individuals werenʼt sampled in both time periods used for temporal changes in genetic structure? E.g., in Pescadero thereʼs only 6 years between sampling periods, presumably these garters are living 6+ years and the small population sizes at these sites might make it more likely to sample the same individuals after six years.

**Response to Reviewer 2: For most sites, the time between tissue sample collection was greater than 10 years, and as Reviewer 2 noted, only Pescadero was shorter at 6 years. We do not know the actual generation time of *Thamnophis sirtalis* but an average generation time for gartersnakes is approximately 3-4 years. Only a limited number of studies on survival in T. s. tetrataenia have been conducted, but they have shown that under ideal conditions only 50% of neonates survive after 2 years of age and only 2% of neonates survive to age 5 (Barry 1996). So our temporal samples are likely sampling different generations with a low likelihood of individuals present in both temporal samples. In addition, tissue samples were taken using tail tips and if a snake was captured with a blunt tail, then this was a good indication that a tissue sample had already been collected. Additionally, as mentioned in the Methods section we marked individuals at all sites with a unique ventral brand and used Passive Integrated Transponders (PIT) tags. When an individual was recaptured, we would not take a second tissue sample. Lastly, we did look for duplicate genotypes in our dataset and did not find any.**

Were these tissue samples accessioned at a natural history collection? I really like the temporal sampling, and in the discussion the authors state that future studies could build on this study but if a different approach to generating sequence data was used it wouldnʼt be possible. Perhaps I missed it, but I would really like to see these samples available.

**Response to Reviewer 2: The San Diego Genetic Facility at USGS houses genetic materials that are part of active scientific investigations. The Smithsonian is the official repository for USGS samples upon completion of active research. The Sundry Civil Act of March 3, 1879 (20 U.S.C. 59), as amended, directs that “All collections of rocks, minerals, soils, fossils, and objects of natural history, archaeology, and ethnology, made by the National Ocean Survey, the [United States] Geological Survey, or by any other parties for the Government of the United States, when no longer needed for investigations in progress shall be deposited in the National Museum [Smithsonian Institution National Museum of Natural History].**
